# Rocky reef biodiversity survey: Punta Pardelas, Argentina

**DOI:** 10.3897/BDJ.9.e72081

**Published:** 2021-12-20

**Authors:** Gonzalo Bravo, Juan Pablo Livore, Nicolás Battini, Marianela Gastaldi, Daniel Lauretta, Martín Brogger, María Paula Raffo, Cristian Lagger, Gregorio Bigatti

**Affiliations:** 1 Instituto de Biología de Organismos Marinos (IBIOMAR), CCT CONICET- CENPAT, Puerto Madryn, Argentina Instituto de Biología de Organismos Marinos (IBIOMAR), CCT CONICET- CENPAT Puerto Madryn Argentina; 2 Fundación ProyectoSub, Puerto Madryn, Argentina Fundación ProyectoSub Puerto Madryn Argentina; 3 Escuela Superior de Ciencias Marinas - Universidad Nacional del Comahue, San Antonio Oeste, Argentina Escuela Superior de Ciencias Marinas - Universidad Nacional del Comahue San Antonio Oeste Argentina; 4 Museo Argentino de Ciencias Naturales Bernardino Rivadavia, Buenos Aires, Argentina Museo Argentino de Ciencias Naturales Bernardino Rivadavia Buenos Aires Argentina; 5 Centro Para el Estudio de Sistemas Marinos (CESIMAR)- CCT CONICET- CENPAT, Puerto Madryn, Argentina Centro Para el Estudio de Sistemas Marinos (CESIMAR)- CCT CONICET- CENPAT Puerto Madryn Argentina; 6 Consejo Nacional de Investigaciones Científicas y Técnicas (CONICET)- Instituto de Diversidad y Ecología Animal (IDEA), Córdoba, Argentina Consejo Nacional de Investigaciones Científicas y Técnicas (CONICET)- Instituto de Diversidad y Ecología Animal (IDEA) Córdoba Argentina; 7 Universidad Nacional de Córdoba, Facultad de Ciencias Exactas, Físicas y Naturales. Laboratorio de Ecología Marina,, Córdoba, Argentina Universidad Nacional de Córdoba, Facultad de Ciencias Exactas, Físicas y Naturales. Laboratorio de Ecología Marina, Córdoba Argentina; 8 Universidad Espíritu Santo, Guayaquil, Ecuador Universidad Espíritu Santo Guayaquil Ecuador

**Keywords:** sampling event, rocky reef, Southern Ocean, photoquadrats, biodiversity, scientific diving

## Abstract

**Background:**

Temperate rocky reefs in the SW Atlantic are productive areas that support highly diverse communities of invertebrates, algae and fishes. Rocky outcrops form complex structures which offer a diversity of microhabitats that lead to a great variety of co-existing species. Subtidal biodiversity within the Natural Protected Area Península Valdés is largely unexplored and studies are mainly limited to fish. A total of 560 high definition photoquadrats from seven rocky reefs (1-25 m depth) at Punta Pardelas were obtained during March 2019. In total, 4491 occurrences were recorded and identified to phyla (n = 2), superclasses (n = 1), classes (n = 5), subclasses (n = 2), orders (n = 2), families (n = 1), subfamilies (n = 1), genera (n = 10) and species (n = 43) levels. This dataset was developed to provide a baseline inventory of Punta Pardelas inside the Natural Protected Area, that was only partially reported more than 50 years ago. Such data represent the first step towards monitoring these less-accessible ecosystems.

**New information:**

Most of the available information about Atlantic Patagonian marine biodiversity is related to rocky intertidal communities or rocky reef fish communities. Despite having more than 4000 km of coastline, in the last 20 years only four studies have focused on subtidal benthic communities from shallow rocky reefs in Argentina (Genzano et al. 2011, Rechimont et al. 2013, Bravo et al. 2015, Bravo et al. 2020a). However, none of them described the epi-benthic community of different surface orientations on the rocky reefs. This dataset includes several surface orientations (i.e. horizontal, vertical, overhang and cave floor) and their microhabitats. We found almost double the number of taxa previously reported for the area. Through stratified sampling of different surface orientations, we recorded species that are often overlooked and thus registered as part of the existing biodiversity. For example, overhang surfaces in our study showed a unique assemblage and a great diversity of sponges. This work will be valuable as baseline information that is currently out of date in Nuevo Gulf rocky reefs.

## Introduction

As in most parts of the world, in Patagonia Argentina, there is more information about biodiversity in intertidal than subtidal habitats (Miloslavich et al. 2011, Rechimont et al. 2013). This region contains large areas where underwater marine life remains largely unexplored (Bigatti and Signorelli 2018), even in easily accessible shallow diving locations. Detecting changes in biodiversity with the current gaps in baseline data may be difficult or even impossible (Fraschetti et al. 2008, Halpern et al. 2008, Duffy et al. 2013). There is, therefore, an urgent need to acquire data of under-sampled areas in order to collect baseline information, monitor and detect changes in species composition due to environmental or anthropogenic processes.

Rocky reefs are an important component of the coastal subtidal ecosystems of Atlantic Patagonia in Argentina. They create a unique habitat that is distinguished from rocky flat or soft bottoms because of the presence of outcrops with crevices and small caves that provide refuges for fish species that are only found in these systems (Galván et al. 2009). As such, rocky reefs provide important marine ecosystem services for recreational fishing, scuba diving activities and have educational and scientific research value (Bravo et al. 2015, Bravo et al. 2020).

Targeting these habitats for subtidal monitoring programmes is essential to detect changes that may occur in the future due to rising sea-water temperature, extreme weather events, marine heat waves or other environmental or human stressors. This study provides new benthic biodiversity baseline data from Punta Pardelas, inside a Natural Protected Area where a single study was performed 55 years ago (Olivier et al. 1966). According to information gathered during this sampling and comparing with previous sudies in Nuevo Gulf (Olivier et al. 1966, Bravo et al. 2015), we conclude that the area is a local biodiversity hotspot and we recommend promoting long-term monitoring in the region.

## Project description

### Title

“Biodiversidad bentónica de arrecifes rocosos de la costa patagónica: estado actual y predicciones ante futuros escenarios de cambio climático” [Biodiversity of epi-benthic communities along template rocky reefs in the Patagonian Atlantic Coast: current state and modelling under a climate change scenario]

### Personnel

Gonzalo Bravo, Juan Pablo Livore, Gregorio Bigatti, Nicolás Battini, Marianela Gastaldi, Daniel Lauretta, Martín Brogger, María Paula Raffo, Cristian Lagger

### Study area description

**Figure 1.**
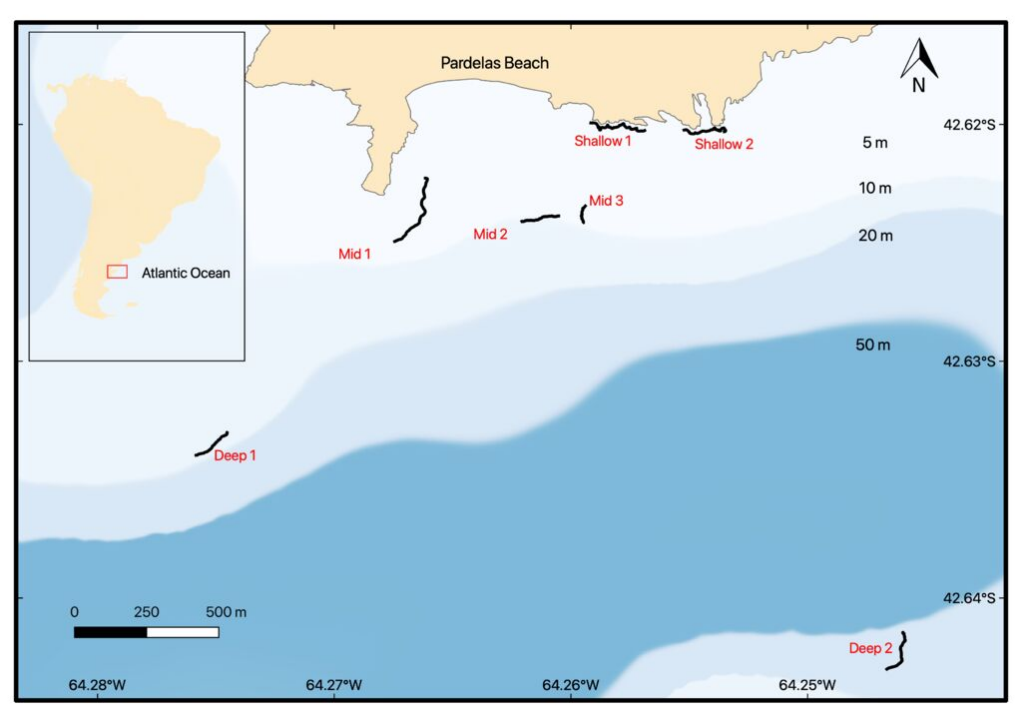
Study site, location and extension of all the rocky reefs sampled. Black lines represented the GPS track of the rocky reefs ledges.

Seven rocky reefs, grouped in an area of about 11 km^2^, were sampled off the coast of Punta Pardelas Bay inside Nuevo Gulf, Atlantic Patagonia (Fig. 1). The study area is part of the Natural Protected Area Península Valdés and this work was performed with the corresponding permit provided by the Subsecretaría de Conservación y Áreas Protegidas (DISPOSICIÓN N°076-SsCyAP/18) from Chubut Government. This latitude is considered as an ecotone of two marine biogeographic provinces (Argentinian and Magellanic), with both warm temperate and cold temperate species represented (Balech and Ehrlich 2008). The tidal regime is semi-diurnal with mean amplitudes of 3.8 m and spring tides of up to 5.7 m. Water temperature varies from 9-18°C. The first and only study of the subtidal benthic community at Punta Pardelas was performed by Olivier et al. (1966), but only down to 10 m depth. Our dataset presents species occurrences and species richness taken by underwater photoquadrats. Sessile species were recorded as percentage cover and mobile fauna as density. It is likely that some taxa, particularly mobile fauna, may present an avoidance behaviour and, thus, was not recorded. Hence, biodiversity estimation is likely underestimated through this methodology. However, we are confident that, for benthic reef biodiversity surveys, it is a cost-effective method and we encourage its use.

### Design description

**Figure 2.**
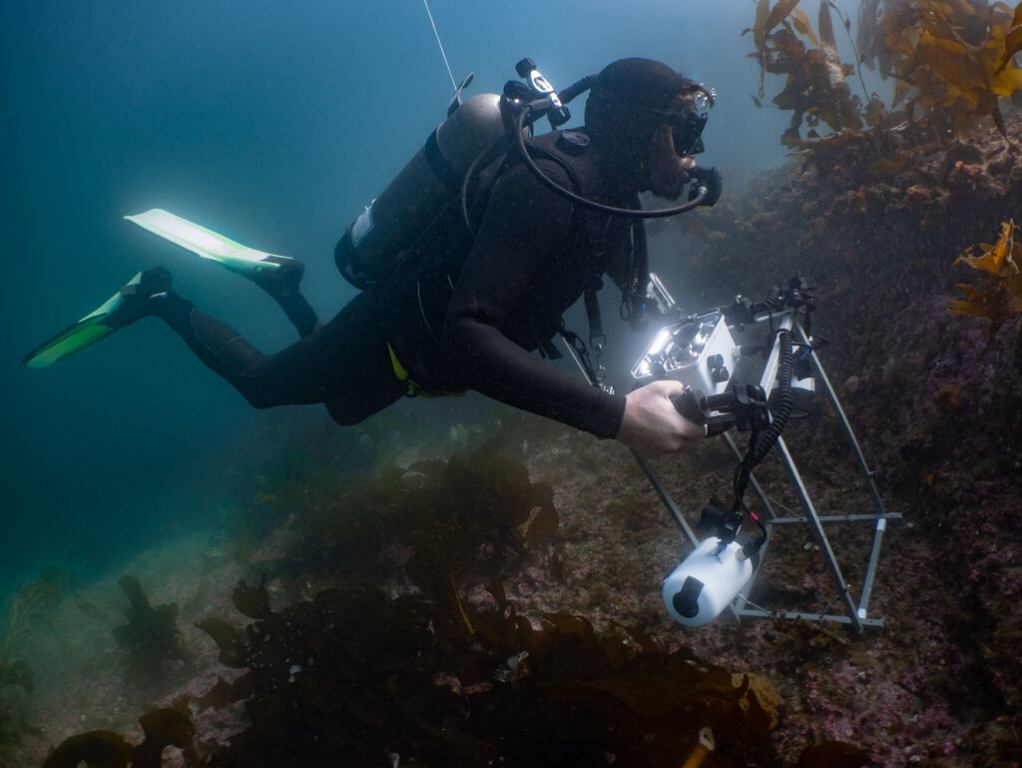
Diver with photoquadrat frame (25 x 25 cm) and GPS line. Photo: Yann Herrera Fuchs.

Ledge borders were followed as underwater transects in all rocky reefs. Photoquadrats (25 x 25 cm), spaced at 2-5 m intervals, were taken by scuba diving (Fig. 2). Preliminary tests showed that a focal length of 50 cm, which, in turn, determined quadrat size, was the best to reduce the negative influence of water turbidity on the resolution of the image. The presence of cavities with a height of 1.5-3.0 m below the rocky ledges provided enough space to sample four different surface orientations (horizontal, vertical, overhang and cave floor). Rocky reefs were sampled at three different depths ranges 1-7 m: “shallow rocky reefs” (n = 2 reefs), 8-15 m: “mid-depth rocky reefs” (n = 3 reefs) and 16-25 m: “deep rocky reefs” (n = 2 reefs). Voucher samples were collected to confirm photo identification when necessary.

### Funding

The major part of the financial support came from PICT-2018-0969 (ANPCyT- ARGENTINA). Minor funding was provided by a Rapid Ocean Conservation grant (ROC) from Waitt Foundation (https://www.waittfoundation.org/), Tides Foundation Grant Award TF2002-089196, Instituto de Conservación de Ballenas (ICB) with the Australis award granted to Gonzalo Bravo and ProyectoSub Foundation. All the authors are members of national institutions in Argentina.

## Sampling methods

### Study extent

This dataset presents species occurrences and species richness of underwater photoquadrats over rocky reefs in Punta Pardelas, Nuevo Gulf. Sessile species were recorded as percentage cover and mobile fauna as density. This is the first study of benthic communities in Punta Pardelas at three different depth levels: shallow rocky reefs (1-7 m), mid-depth rocky reefs (8-15 m) and deep rocky reefs (16-25 m).

### Sampling description

Divers were equipped with a Canon 100D camera and two Ikelite DS-161 strobes, mounted on a stainless-steel structure with a 0.0625 m^2^ quadrat (0.25 x 0.25 m). The camera had a 18-55 mm Canon lens and all the images were taken with the 18 mm setting, autofocus, ISO 400, Exposure 1/200 s at f/11 and flashes set on automatic TTL. A dive computer (Oceanic Geo2) was mounted on one side of the quadrat to register the depth and temperature of each photoquadrat. Divers carried a monofilament line that towed a surface buoy with a GPS loading a waypoint every 3 seconds (Bravo et al. 2021).

### Quality control

Species names were assigned when the photograph allowed us to observed the taxonomic diagnostic features of the organisms. When identification was inconclusive, only family or genus names were assigned and, in the case of filamentous algae or sponges, functional groups were assigned. Most of the species were identified by the project co-director Gonzalo Bravo who has extensive knowledge and observations of the local species in the field (see: https://www.inaturalist.org/lifelists/gonzalobravopatagonia). In some cases, extractive samples were collected for taxonomic confirmation or description of species not recorded in the area. The taxonomists who contributed to the identification of photoquadrats and extractive samples were Paula Raffo (Algae), Marianela Gastaldi (Porifera), Cristian Lagger (Tunicates), Martín Brogger (Echinodermata), Gregorio Bigatti (Mollusca) and Daniel Lauretta (Cnidaria: Actiniaria, Corallimorpharia). The taxonomic validity of the names was verified using the World Register of Marine Species (WoRMS; www.marinespecies.org). The geo-referencing of photoquadrats was recorded using a Garmin eTrex 10 GPS (WGS84 Datum) with a 5 m accuracy.

### Step description

- GPS and underwater camera time were synchronised. This was done by aligning the camera clock with the GPS clock before each dive. The GPS was set on track mode recording one waypoint every 3 seconds.

- The portable GPS (Garmin Etrex 10) was placed in a dry bag on top of a Rescue Can buoy connected to the diver by a monofilament line using a diving reel. Divers maintained the monofilament line as tightly as possible to minimise angles between the buoy and the diver.

- Photoquadrat sampling.

- Photos were georeferenced using the function “Auto-tag photos” in Adobe Lightroom Classic version: 9.1.

- Percentage cover of algae and sessile invertebrates was calculated using a 100 point grid overlaid on each photo, using CoralNet software (Beijbom et al. 2015). On the same image, all the mobile fauna was counted to calculate density. All the photoquadrats are stored in a public CoralNet source: https://coralnet.ucsd.edu/source/1933/

## Geographic coverage

### Description

Nuevo Gulf in Chubut Province, Argentina. We selected seven rocky reefs grouped in an area of almost 11 km^2^ in Punta Pardelas.

### Coordinates

-42.652 and -42.617 Latitude; -64.284 and -64.225 Longitude.

## Taxonomic coverage

### Description

**Figure 3.**
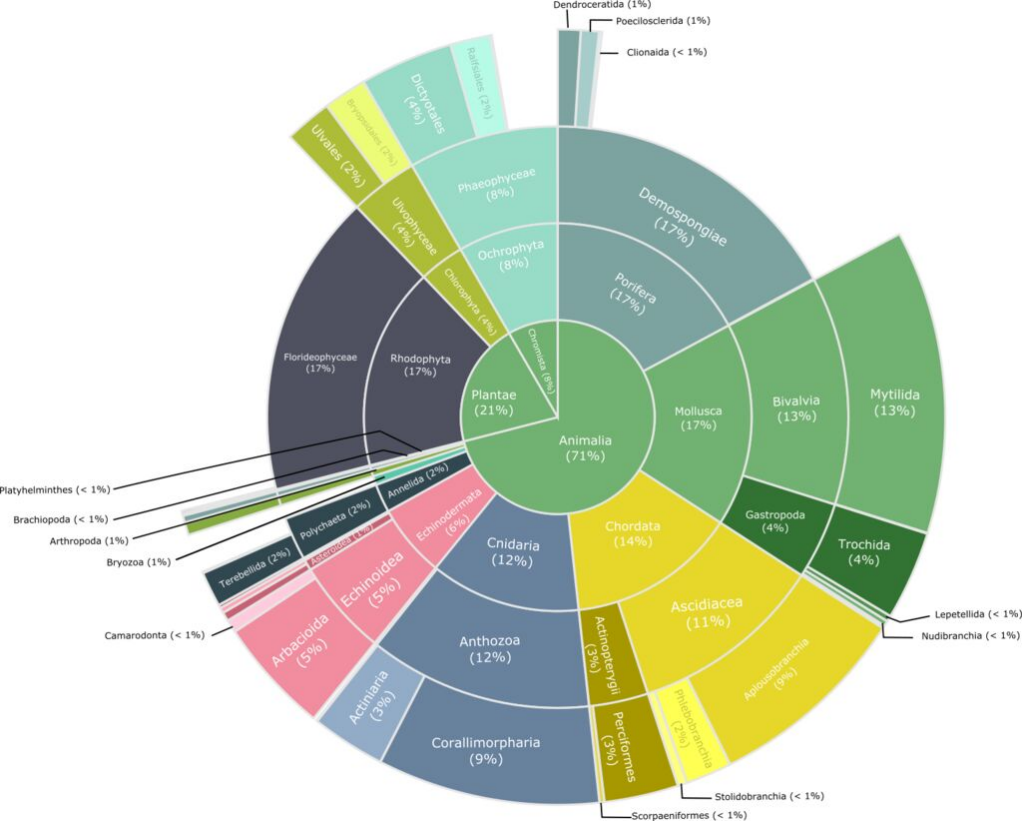
Taxonomic distribution and coverage.

The database by Bravo et al. (2020b), described here, is based on photoquadrat taxonomic identification and is supported by local taxonomists. The goal of this study was to update the benthic community data from Punta Pardelas in Nuevo Gulf, Atlantic Patagonia. The taxonomic coverage (Fig. 3) includes twelve phyla: Porifera (17%), Mollusca (17%), Rhodophyta (17%), Chordata (14%), Cnidaria (12%), Ochrophyta (8%), Echinodermata (6%), Chlorophyta (4%), Annelida (2%), Arthropoda (1%), Brachiopoda (< 1%) and Phatyhelminthes (< 1%). The class Calcarea (Porifera), the genus Halcurias (Cnidaria: Actiniaria) and the species *Darwinellarosacea* (Porifera) represented first records for Nuevo Gulf and were identified by extractive samples by taxonomists.

### Taxa included

**Table:** 

Rank	Scientific Name	
species	*Ascidiellaaspersa*	
species	*Aulacomyaatra*	
species	*Anthothoechilensis*	
genus	*Aplidium*	
species	*Austromegabalanuspsittacus*	
species	*Asterocarpahumilis*	
species	*Corellaeumyota*	
species	*Aequipectentehuelchus*	
species	*Magellaniavenosa*	
phylum	Bryozoa	
species	*Corynactiscarnea*	
species	*Cionaintestinalis*	
order	Aplousobranchia	
species	*Cionarobusta*	
subfamily	Lithophaginae	
species	*Diplosomalisterianum*	
genus	*Halcurias*	
class	Hydrozoa	
species	*Lissoclinumfragile*	
species	*Metridiumsenile*	
genus	*Myxicola*	
species	*Paramolgulagregaria*	
species	*Parabunodactisimperfecta*	
genus	*Clathria*	
genus	*Cliona*	
class	Demospongiae	
species	*Darwinellarosacea*	
species	*Tripaleaclavaria*	
family	Terebellidae	
class	Polychaeta	
order	Ralfsiales	
superclass	Corallinophycidae	
genus	*Codium*	
species	*Corallinaofficinalis*	
species	*Colpomeniasinuosa*	
species	*Dictyotadichotoma*	
subclass	Rhodymeniophycidae	
species	*Lomentariaclavellosa*	
class	Phaeophyceae	
genus	*Ulva*	
species	*Undariapinnatifida*	
class	Calcarea	
species	*Pseudechinusmagellanicus*	
species	*Tegula patagonica*	
species	*Leucippapentagona*	
species	*Arbaciadufresnii*	
genus	*Patagonotothen*	
species	*Ribeiroclinuseigenmanni*	
species	*Helcogrammoidescunninghami*	
species	*Pachycheleschubutensis*	
species	*Dorisfontainii*	
species	*Diaululapunctuolata*	
species	*Allostichastercapensis*	
species	*Polyceramarplatensis*	
species	*Fissurellideapatagonica*	
species	*Fissurellaradiosatixierae*	
species	*Phrikocerosmopsus*	
species	*Cosmasteriaslurida*	
species	*Sebastesoculatus*	
genus	*Trapania*	
species	*Cycethraverrucosa*	
species	*Pleurobranchaeamaculata*	
species	*Ophioplocusjanuarii*	
species	*Odontasterpenicillatus*	
genus	*Calliostoma*	
subclass	Heterobranchia	

## Traits coverage

### Data coverage of traits

PLEASE FILL IN TRAIT INFORMATION HERE

## Temporal coverage

### Notes

2019-03-11 through 2019-03-26

## Usage licence

### Usage licence

Creative Commons Public Domain Waiver (CC-Zero)

### IP rights notes

This work is licensed under a Creative Commons Attribution (CC-BY) 4.0 License.

## Data resources

### Data package title

Rocky Reef Biodiversity Survey: Punta Pardelas, Argentina

### Resource link

https://www.gbif.org/dataset/16a62f7b-e52d-49b6-8605-b69d22d0572c

### Alternative identifiers

http://arobis.cenpat-conicet.gob.ar:8081/resource?r=arrs

### Number of data sets

1

### Data set 1.

#### Data set name

Rocky Reef Biodiversity Survey: Punta Pardelas, Argentina

#### Data format

Darwin Core

#### Number of columns

40

#### Download URL

http://arobis.cenpat-conicet.gob.ar:8081/resource?r=arrs#downloads

**Data set 1.** 

Column label	Column description
eventID	An identifier for the set of information associated with an Event (something that occurs at a place and time). May be a global unique identifier or an identifier specific to the dataset.
occurrenceID	Is an identifier for the occurrence record and should be persistent and globally unique.
parentEventID	An identifier for the broader Event that groups this and potentially other Events.
measurementType	The nature of the measurement, fact, characteristic or assertion.
measurementTypeID	An identifier for the measurementType (global unique identifier, URI). The identifier should reference the measurementType in a vocabulary.
measurementValue	The value of the measurement, fact, characteristic or assertion.
measurementUnit	The units associated with the measurementValue.
measurementUnitID	An identifier for the measurementUnit (global unique identifier, URI). The identifier should reference the measurementUnit in a vocabulary.
eventDate	The date and time at which an occurrence was recorded (This term uses the ISO 8601 format).
Year	The four-digit year in which the Event occurred, according to the Common Era Calendar.
country	The name of the country or major administrative unit in which the Location occurs.
countryCode	The standard code for the country in which the Location occurs (ISO 3166-1- alpha-2 country code).
stateProvince	The name of the next smaller administrative region than country (state, province, canton, department, region etc.) in which the Location occurs.
locality	The specific description of the place (wide-ranging).
site	The specific description of the place (narrow).
decimalLongitude	The geographic longitude (in decimal degrees, using the spatial reference system given in geodeticDatum) of the geographic centre of a Location. Positive values are east of the Greenwich Meridian, negative values are west of it. Legal values lie between -180 and 180, inclusive.
decimalLatitude	The geographic latitude (in decimal degrees, using the spatial reference system given in geodeticDatum) of the geographic centre of a Location. Positive values are north of the Equator, negative values are south of it. Legal values lie between -90 and 90, inclusive.
coordinateUncertaintyInMetres	The horizontal distance (in metres) from the given decimalLatitude and decimalLongitude describing the smallest circle containing the whole of the Location. Leave the value empty if the uncertainty is unknown, cannot be estimated or is not applicable (because there are no coordinates). Zero is not a valid value for this term.
geodeticDatum	The ellipsoid, geodetic datum or spatial reference system (SRS) upon which the geographic coordinates given in decimalLatitude and decimalLongitude are based.
minimumDepthInMetres	The lesser depth of a range of depth below the local surface, in metres.
maximumDepthInMetres	The greater depth of a range of depth below the local surface, in metres.
sampleSizeValue	A numeric value for a measurement of the size (time duration, length, area or volume) of a sample in a sampling event.
sampleSizeUnit	The unit of measurement of the size (time duration, length, area or volume) of a sample in a sampling event.
institutionCode	Identifies the custodian institute (often by acronym).
basisOfRecord	The specific nature of the data record.
recordedBy	A person, group or organisation responsible for recording the original Occurrence.
scientificName	The full scientific name, with authorship and date information, if known. When forming part of an Identification, this should be the name in lowest level taxonomic rank that can be determined.
scientificNameID	An identifier for the nomenclatural (not taxonomic) details of a scientific name.
taxonID	An identifier for the set of taxon information (data associated with the Taxon class). May be a global unique identifier or an identifier specific to the dataset.
acceptedNameUsage	The full name, with authorship and date information if known, of the currently valid (zoological) or accepted (botanical) taxon.
scientificNameAuthorship	The authorship information for the scientificName formatted according to the conventions of the applicable nomenclaturalCode.
kingdom	The full scientific name of the kingdom in which the taxon is classified.
phylum	The full scientific name of the phylum or division in which the taxon is classified.
class	The full scientific name of the class in which the taxon is classified.
order	The full scientific name of the order in which the taxon is classified.
family	The full scientific name of the family in which the taxon is classified.
genus	The full scientific name of the genus in which the taxon is classified.
subgenus	The full scientific name of the subgenus in which the taxon is classified.
specificEpithet	The name of the first or species epithet of the scientificName.
infraspecificEpithet	The name of the lowest or terminal infraspecific epithet of the scientificName, excluding any rank designation.
